# Dataset of calcified plaque condition in the stenotic coronary artery lesion obtained using multidetector computed tomography to indicate the addition of rotational atherectomy during percutaneous coronary intervention

**DOI:** 10.1016/j.dib.2016.02.052

**Published:** 2016-02-27

**Authors:** Yasushi Akutsu, Yuji Hamazaki, Teruo Sekimoto, Kyouichi Kaneko, Yusuke Kodama, Hui-Ling Li, Jumpei Suyama, Takehiko Gokan, Koshiro Sakai, Ryota Kosaki, Hiroyuki Yokota, Hiroaki Tsujita, Shigeto Tsukamoto, Masayuki Sakurai, Takehiko Sambe, Katsuji Oguchi, Naoki Uchida, Shinichi Kobayashi, Atsushi Aoki, Youichi Kobayashi

**Affiliations:** a Division of Cardiology, Department of Medicine, Showa University School of Medicine, Japan; b Department of Radiology, Showa University School of Medicine, Japan; c Department of Pharmacology, Showa University School of Medicine, Japan; d Department of Internal Medicine (Cardiology), Clinical Research Institute for Clinical Pharmacology & Therapeutics, Showa University Karasuyama Hospital, Japan; e Department of Cardiovascular Surgery, Showa University School of Medicine, Japan

**Keywords:** Coronary artery calcium scores, Multidetector computed tomography, CT angiography, Rotational atherectomy, Percutaneous coronary intervention

## Abstract

Our data shows the regional coronary artery calcium scores (lesion CAC) on multidetector computed tomography (MDCT) and the cross-section imaging on MDCT angiography (CTA) in the target lesion of the patients with stable angina pectoris who were scheduled for percutaneous coronary intervention (PCI). CAC and CTA data were measured using a 128-slice scanner (Somatom Definition AS+; Siemens Medical Solutions, Forchheim, Germany) before PCI. CAC was measured in a non-contrast-enhanced scan and was quantified using the Calcium Score module of SYNAPSE VINCENT software (Fujifilm Co. Tokyo, Japan) and expressed in Agatston units. CTA were then continued with a contrast-enhanced ECG gating to measure the severity of the calcified plaque condition. We present that both CAC and CTA data are used as a benchmark to consider the addition of rotational atherectomy during PCI to severely calcified plaque lesions.

**Specifications Table**

**Table:** 

Subject area	*Cardiology*
More specific subject area	*Multidetector computed tomography (MDCT)*
Type of data	*Figures*
How data was acquired	*128-slice scanner (Somatom Definition AS+; Siemens Medical Solutions, Forchheim, Germany)*
Data format	*Analyzed data*
Experimental factors	*Regional coronary artery calcium scores (CAC) and cross-section imaging of the coronary artery on MDCT imaging*[1]
Experimental features	*CAC was measured in a non–contrast-enhanced scan and was quantified using the Calcium Score module of SYNAPSE VINCENT software (Fujifilm Co. Tokyo, Japan). MDCT angiography was performed with a contrast-enhanced ECG gating to measure the calcified plaque condition.*
Data source location	*Tokyo, Japan*
Data accessibility	*Data are provided with this article*

**Value of the data**

• Regional CAC obtained from a non-contrast-enhanced multidetector computed tomography (MDCT) imaging is useful for evaluating regional calcified plaque condition because of a simple marker when the percutaneous coronary intervention (PCI) was scheduled in patients with stenotic coronary artery.
• Cross-section imaging of a contrast-enhanced ECG gating MDCT angiography (CTA) is also useful for evaluating the regional calcified plaque condition into the lumen of coronary artery because of a viewpoint from the inside practically when the PCI was scheduled in patients with stenotic coronary artery.
• Both CAC and CTA cross-section imaging data are used as a benchmark to consider the addition of rotational atherectomy during PCI to severely calcified plaque lesions.

## 1 Data

In this present data [1], we provide the total CAC in each patient, vessel CAC of coronary artery with PCI and lesion CAC of the target lesion with PCI on non-contrast-enhanced MDCT imaging in patients with PCI to the stenotic coronary artery. Furthermore, we provide the calcified plaque condition on the cross-sectional and longitudinal imaging of MDCT angiography in patients with PCI to the stenotic coronary artery.

## 2 Experimental design, materials and methods

### 2.1 Patients

The data of CAC and CTA on MDCT were obtained in patients with stable angina pectoris who were scheduled for first PCI.

### 2.2 Collection of CAC and CTA data

CAC data were obtained using a non-contrast-enhanced 128-slice scanner (Somatom Definition AS+; Siemens Medical Solutions, Forchheim, Germany) before PCI. CAC data was quantitatively analyzed using the Calcium Score module of SYNAPSE VINCENT software (Fujifilm Co. Tokyo, Japan, http://www.fujifilmusa.com/products/medical/radiology/3d/) [2] and was expressed in Agatston units [3]. CTA data was obtained using a contrast-enhanced ECG gating scanner (Omnipaque, 350 mg iodine/mL, Daiichi Sankyo Co, Ltd., Tokyo, Japan). A retrospective, ECG-gated acquisition protocol was used in all patients, with collimation width, 2×64×0.6 mm; rotation time, 300 ms; tube voltage, 120 kV; effective tube current, 800 mA; table feed, 11.5 mm/rotation; and pitch, 0.3. The raw CT data were reconstructed using algorithms optimized for retrospective ECG-gated segment reconstruction with a 0.6-mm slice thickness and a 0.3 mm increment. The optimal cardiac phase displaying the minimum motion artifact was individually determined.

### 2.3 Analysis of CAC data

**Fig. 1:**
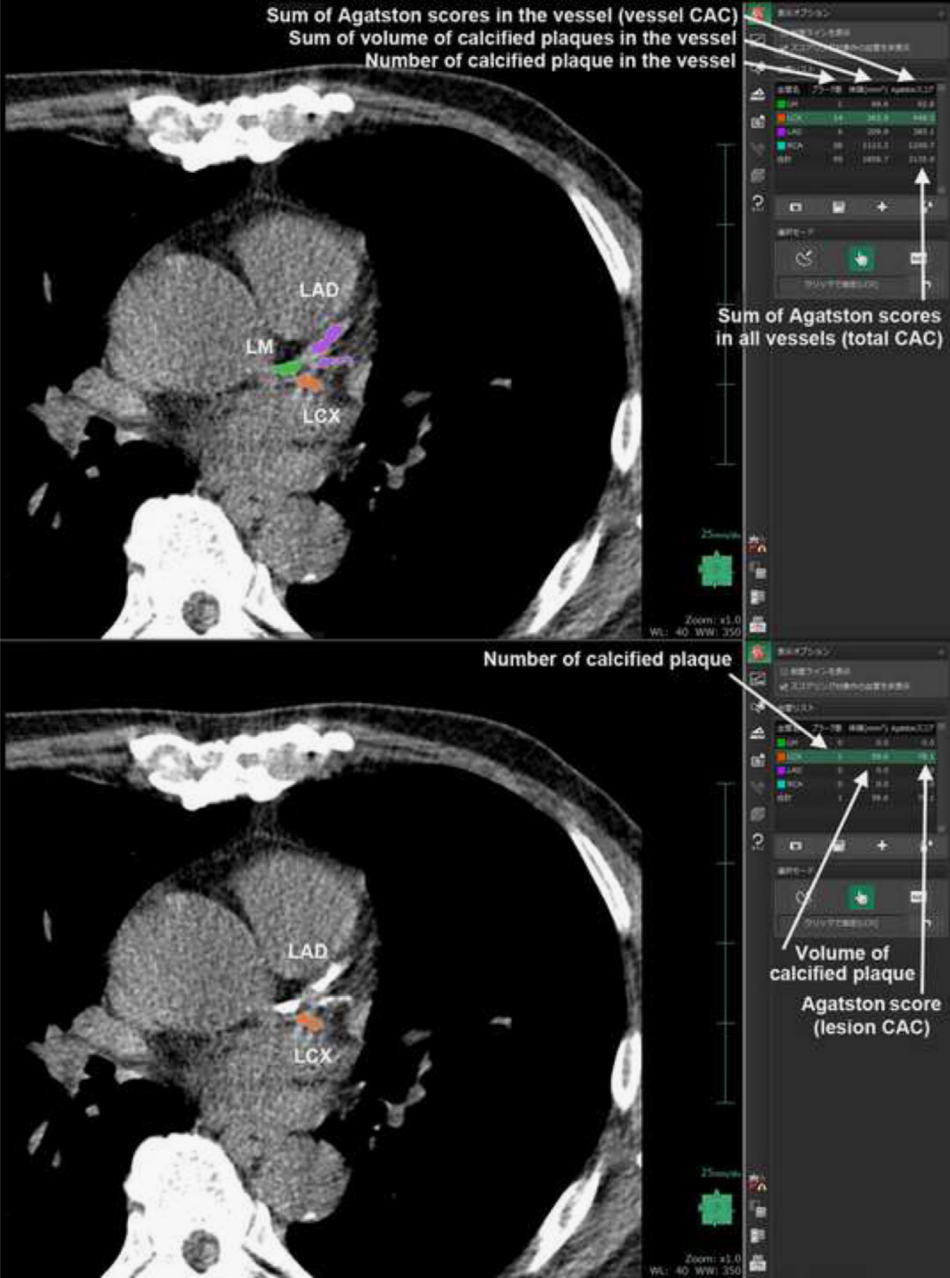
The calcified plaque lesions were selected automatically, and was displayed by color, and the calcium scoring was calculated using Agatston score (total CAC, vessel CAC, and lesion CAC).

The software displayed the calcified plaque lesion of the coronary artery by color and calculated the quantitative value of the calcified plaque by using the Agatston score method (Fig. 1). CAC data was measured for each patient (total CAC), vessel (vessel CAC), and target lesion (lesion CAC). CAC was defined as a plaque of the least three contiguous pixels with a density of >130 Hounsfield units (HU). Lesion CAC data was calculated by multiplying the target lesion area by a density factor derived from the maximal HU within this area, as described by Agatston, et al. Total CAC data was determined by summing individual lesion scores from each of the four main coronary arteries (left main coronary, left anterior descending coronary, left circumflex coronary, and right coronary arteries).

### 2.4 Analysis of CTA data

**Fig. 2:**
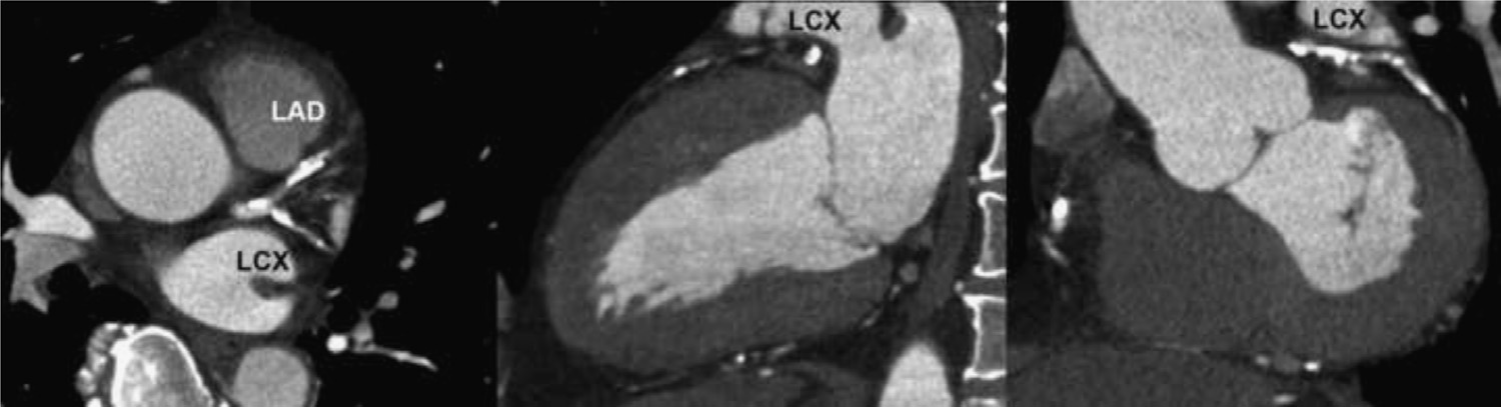
Displays of axial, sagittal, and coronal images showed the multiple calcified plaque lesions in all coronary arteries.

**Fig. 3:**
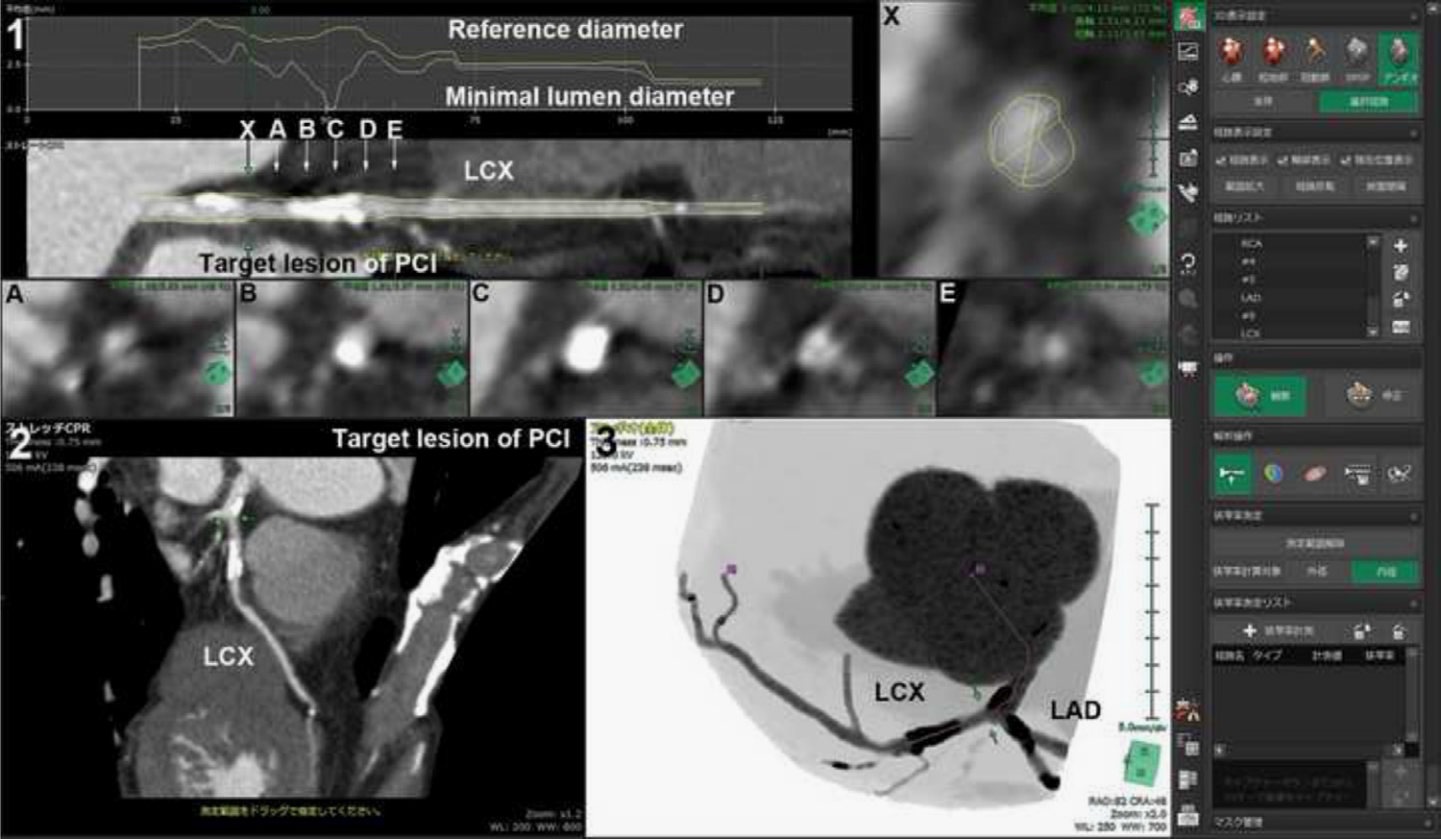
(1) The straight curved planar reconstructions (CPR) image, and**:** the cross-section multiplanar reconstruction (MPR) image at each point of X, and A to E, (2) the stretch CPR image, (3) the angiographic view image by the analytical virtual 3-dimensional model. The severity of coronary artery stenosis and the length of one coronary lesion with ≥75% luminal diameter stenosis were measured by the straight CPR image and the cross-section MPR image. The severity of the calcified plaque condition was assessed using the number of quadrants involving calcium on an arterial cross-section (MPR).

The software extracted the path of the target blood vessels (Fig. 2) and visualized the straight and stretch curved planar reconstructions (CPR) images, and the cross-section multiplanar reconstruction (MPR) image [4] (Fig. 3). The data of target lesion length that was measured on the longitudinal images using CPR was defined as the extent of one coronary lesion with ≥75% luminal diameter stenosis of the coronary artery on CTA corresponding to the target lesion of PCI on CAG. The data of calcified plaque condition was assessed using the number of quadrants involving calcium on an arterial cross-section (MPR) as follows: (grade 1) non-calcified (no calcification); (grade 2) one cross-section calcification arc <90° (grade 3) one calcification arc ≥90 and <180 or multiple calcifications on cross-section with residual lumen visible; (grade 4) one calcification arc ≥180 and <270; and (grade 5) one calcification arc ≥270 or without residual lumen visible according to a previous study [5]. The data of calcified plaque pattern in the target lesion was defined as the higher grade of calcium plaque patterns within this area.
