# Supplementary material for: Dataset of calcified plaque condition in the stenotic coronary artery lesion obtained using multidetector computed tomography to indicate the addition of rotational atherectomy during percutaneous coronary intervention
Source: Data Brief. 2016 Feb 27;7:376–80. doi: 10.1016/j.dib.2016.02.052 (PMC4781927; doi:10.1016/j.dib.2016.02.052)
Supplement: Supplementary file 1 — Supplementary material [file mmc1.doc]

**Manuscript title:** Dataset of Calcified Plaque Condition in the Stenotic Coronary Artery Lesion obtained using Multidetector Computed Tomography to indicate the Addition of Rotational Atherectomy during Percutaneous Coronary Intervention

**Authors:** Yasushi Akutsu^ad^, Yuji Hamazaki^a^, Teruo Sekimoto^a^, Kyouichi Kaneko^a^, Yusuke Kodama^a^, Hui-Ling Li^a^, Jumpei Suyama^b^, Takehiko Gokan^b^, Koshiro Sakai^a^, Ryota Kosaki^a^, Hiroyuki Yokota^a^, Hiroaki Tsujita^a^, Shigeto Tsukamoto^a^, Masayuki Sakurai^a^, Takehiko Sambe^cd^, Katsuji Oguchi^c^, Naoki Uchida^cd^, Shinichi Kobayashi^cd^, Atsushi Aoki^e^ and Youichi Kobayashi^a^

**Affiliations:**

^a^Division of Cardiology, Department of Medicine, ^b^Department of Radiology, ^c^Department of Pharmacology , Department of Cardiovascular Surgery^e^, Showa University School of Medicine

^d^Department of Internal Medicine (Cardiology), Clinical Research Institute for Clinical Pharmacology & Therapeutics, Showa University Karasuyama Hospital

Contact email: hzn01233@s02.itscom.net

**Conflicts of interest:** none
